# Experiences of Electronic Health Records’ and Client Information Systems’ Use on a Mobile Device and Factors Associated With Work Time Savings Among Practical Nurses: Cross-Sectional Study

**DOI:** 10.2196/46954

**Published:** 2024-05-29

**Authors:** Satu Paatela, Maiju Kyytsönen, Kaija Saranto, Ulla-Mari Kinnunen, Tuulikki Vehko

**Affiliations:** 1 Health and Social Service System Research Finnish Institute for Health and Welfare Helsinki Finland; 2 Faculty of Social Sciences and Business Studies University of Eastern Finland Kuopio Finland; 3 Department of Health and Social Management University of Eastern Finland Kuopio Finland

**Keywords:** practical nurse, information and communication technology, electronic health record, client information system, documentation, mobile technology

## Abstract

**Background:**

The transmission of clinical information in nursing predominantly occurs through digital solutions, such as computers and mobile devices, in today’s era. Various technological systems, including electronic health records (EHRs) and client information systems (CISs), can be seamlessly integrated with mobile devices. The use of mobile devices is anticipated to rise, particularly as long-term care is increasingly delivered in environments such as clients’ homes, where computers are not readily accessible. However, there is a growing need for more user-centered data to ensure that mobile devices effectively support practical nurses in their daily activities.

**Objective:**

This study aims to analyze practical nurses’ experiences of using EHRs or CISs on a mobile device in their daily practice. In addition, it aims to examine the factors associated with work time savings when using EHRs/CISs on a mobile device.

**Methods:**

A cross-sectional study using an electronic survey was conducted in spring 2022. A total of 3866 practical nurses participated in the survey based on self-assessment. The sample was limited to practical nurses who used EHRs or CISs on a mobile device and worked in home care or service housing within the social welfare or health care sector (n=1014). Logistic regression analysis was used to explore the factors associated with work time savings.

**Results:**

The likelihood of perceiving work time savings was higher among more experienced EHR/CIS users compared with those with less experience (odds ratio [OR] 1.59, 95% CI 1.30-1.94). Participants with 0-5 years of work experience were more likely to experience work time savings compared with those who had worked 21 years or more (OR 2.41, 95% CI 1.43-4.07). Practical nurses in home care were also more likely to experience work time savings compared with those working in service housing (OR 1.95, 95% CI 1.23-3.07). A lower grade given for EHRs/CISs was associated with a reduced likelihood of experiencing work time savings (OR 0.76, 95% CI 0.66-0.89). Participants who documented client data in a public area were more likely to experience work time savings compared with those who did so in the nurses’ office (OR 2.33, 95% CI 1.27-4.25). Practical nurses who found documentation of client data on a mobile device easy (OR 3.05, 95% CI 2.14-4.34) were more likely to experience work time savings compared with those who did not. Similarly, participants who believed that documentation of client data on a mobile device reduced the need to memorize things (OR 4.10, 95% CI 2.80-6.00) were more likely to experience work time savings compared with those who did not.

**Conclusions:**

To enhance the proportion of practical nurses experiencing work time savings, we recommend that organizations offer comprehensive orientation and regular education sessions tailored for mobile device users who have less experience using EHRs or CISs and find mobile devices less intuitive to use.

## Introduction

Information and communication technology (ICT) including electronic health records (EHRs) and client information systems (CISs) have become an increasingly important part of health care and social services in Finland [1-4]. In fact, EHR has been a common tool in Finnish health care for a long time, and from 2010 onward EHR availability has been 100% in public and private facilities [5]. EHRs include a comprehensive collection of patient health information (eg, narrative texts and laboratory data), with the collected data used in the care processes of the patient [6]. By contrast, CISs are more commonly used in the public social welfare sector for accessing, storing, and using client information and documents [3,4]. The Finnish Ministry of Social Affairs and Health has been at the helm of guiding the development of technological infrastructure and associated legislative efforts over the decades. Their eHealth and eWelfare strategy aims to improve the effective utilization of data in bolstering service renewal and citizen well-being at a national level [7].

With the development of the ICT infrastructure, the clinical information in nursing is nowadays mainly transmitted via digital solutions such as computers and mobile devices [8]. Particularly, mobile devices such as smartphones and tablets are commonly used in the health care sector [9,10], and nurses are known to use EHRs on mobile devices 3 times more often compared with doctors in a hospital environment [11]. Different technological systems such as EHRs can be integrated with mobile devices [9,11-13], and this allows social and health care professionals to document client data and exchange information related to the clients and service assignments in the system regardless of the time and location [11,13]. Thus, the ability to receive nursing information via a mobile device can promote the mobility and portability of care and enhance service flexibility [9,12].

In previous studies, nurses have been reported to perceive mobile devices as beneficial in their daily practice [14,15]. Mobile device use has been found to support nurses’ workflow processes [11,16-18] as they need to spend less time on clinical documentation [17]. In fact, it is important to recognize that the use of different technological systems such as EHRs has been shown to provide nurses with more time for direct patient care and interprofessional communication [19]. Moreover, mobile devices have been reported to be useful for planning work, handling notes [14], and saving time for nurses [14,20,21]. Mobile devices may also reduce duplicate documentation [13] and potential documentation errors [17,20] because client data can be documented at the time of its occurrence. In addition, improved decision-making is one of the main advantages [9,20]. Mobile devices continuously provide the latest information on the situation of the clients, which can improve safety and the quality of care [14,18]. For instance, in home care settings, workers can review and plan real-time nursing interventions and tasks in the clients’ home environment [13,16] because the daily assignments can be seen directly on the mobile device. The use of mobile devices can also contribute to client empowerment as nurses have easier access to clinical information and they can reply to clients’ questions more readily [17].

By contrast, some challenges have emerged related to the use of EHRs/CISs on a mobile device. According to a recent study, the use of EHRs on a mobile device can negatively affect nurses’ well-being because the use of mobile devices may increase time pressure and stress at work as a result of functional problems and changing information systems [10]. In home care settings, challenges have emerged, particularly concerning technical issues such as usability problems. This is because the information systems used on mobile devices are not always tailored to address the specific requirements of various working environments [22]. This in turn may lead to reduced workflows [22]. Additionally, there is a possibility of poor signal connectivity [18] and increased data security threats [17,18,23] when using mobile devices. Furthermore, some of the recent studies have observed that the use of EHRs itself may negatively impact the quality of communication between nurses and patients because nurses’ attention is more focused on documentation tools such as computers rather than on the patient [24,25]. More generally, the lack of digital competence can affect how different digital tools are adopted in practice [26,27].

The European Union has promoted digitalization in society, including public services, with political consensus through the Digital Decade policy program [28]. The change in the demographic structure especially forces social and health care services to invest even more in technological solutions [29] such as mobile documentation [22]. An aging population increases the need to provide long-term care in home environments [30], and therefore, using EHRs and CISs on a mobile device is expected to grow as computers for professionals are rarely available at the patients’ bedside in the home environment [14]. In the Finnish context, practical nurses often take care of needs related to the activities of daily living, for instance, in home care and service housing. Practical nurses in the social and health care profession are strictly regulated by law in Finland [31,32]. Practical nurses are required to have successfully obtained the Vocational Qualification in Social and Healthcare, which entails accruing 180 competence points [33]. Qualified practical nurses are registered with the National Supervisory Authority for Welfare and Health. They are employed across a diverse spectrum of careers within the social welfare and health care sectors, as well as in early childhood education and schools [34]. Practical nurses are the second largest occupational group in Finland and the largest group in the social welfare and health care sectors in Finland, with 79,800 people working as practical nurses at the end of 2020 [35].

As practical nurses form an important group of professionals, it is justified to study their ways of working and increase our knowledge about their experiences of EHR and CIS use on mobile devices. Some of the previous studies have investigated the use of mobile devices from the perspectives of registered nurses, nursing students, and doctors [10,11,14,15], but there is still limited understanding of the experiences of practical nurses. More user-centered data are needed to ensure that mobile devices fit into the changing clinical practice [18] and to improve health professionals’ workflows in those work environments where mobile devices are commonly used. As patient care becomes increasingly complex [8] and health professionals are constantly required to work more efficiently [36], it is important to study whether mobile devices are as effective tools as they are expected to be in the daily activities of practical nurses [9,11,13,14,16,17].

Consequently, the aim of this study was to analyze practical nurses’ experiences regarding their use of EHRs/CISs on a mobile device in their daily practice in home care and service housing settings in the social welfare and health care sectors. Furthermore, we examined the potential factors associated with work time savings when practical nurses were using EHRs/CISs on a mobile device.

## Methods

### Study Context

Finland is a Nordic welfare state where all citizens have universal access to health care and social welfare services. In the 2000s, long-term care for older people and persons with disabilities in Finland has changed from institutional care to more individualized services [37]. In Finland, long-term care is increasingly provided in service housing or in the home environment under social services. Service housing is available for those citizens who need support living independently. These facilities offer a range of services including meal provision, assistance with personal hygiene, and various health care services [38]. However, most older adults continue to reside in their own homes, where they can access home care services if needed. Home care encompasses health center–based home nursing and home help services [37,38]. Finland has a wide array of EHRs and CISs, which are used across both the health care and social welfare sectors [6].

### Study Design and Sample

This was a cross-sectional study based on an electronic survey. Data were collected in the spring of 2022 over a 3-week period using a convenience sampling method. As of the end of 2020, there were 79,800 practical nurses employed in Finland [35], working across the social welfare and health care sectors, as well as in schools and early childhood education and care. Potential respondents were invited to participate in the survey through an email sent by 2 trade unions: The Finnish Union of Practical Nurses and The Union of Public and Welfare Sectors. The electronic survey was distributed to 54,030 members of the trade unions aged 18-65 years. The cover letter specified the study theme as the use of EHRs and CISs. However, previous studies indicate that not all members of the trade unions use EHRs/CISs in their daily practice. This is because practical nurses in social services may still rely on alternative solutions for documentation [6]. Despite this, 2 reminders were sent to potential participants. Ultimately, 3866 practical nurses responded to the survey, yielding a response rate of 7.16%.

In this study, the inclusion criteria for participation were 2-fold: (1) respondents must work as practical nurses and use an EHR or CIS, and (2) they must not be employed in school or early childhood education and care settings. These criteria were outlined in the first 2 questions of the survey, and the survey was closed for potential respondents who did not meet these criteria. The analysis was additionally narrowed down to practical nurses who indicated that they use EHRs or CISs on a mobile device and are employed in either home care or service housing settings (n=1014). Respondents working in other employment settings were excluded because of the limited number of mobile device users in those settings.

### Instrument

The experiences of EHR systems among physicians were initially assessed in Finland through a national survey in 2010 [39,40]. Subsequently, the survey was refined and conducted again in 2014, 2017, and 2021 for physicians. Additionally, it was customized for registered nurses in 2017 [41-43] and for social care professionals (educated at a university or a university of applied sciences) in 2020 [44,45]. Since 2014, these national surveys have been carried out as part of the “Monitoring and Assessment of Social Welfare and Health Care Information System Services” (STePS) projects [6]. In a significant development, for the first time in 2022, the survey was customized and conducted for practical nurses as well. Before data collection, the survey underwent pretesting with 20 practical nurses. Questions regarding the use of EHRs and CISs on mobile devices were particularly emphasized, given their integral role in the workflow of many practical nurses. As a result, this study specifically centered on the utilization of a mobile device for the documentation of client data.

A total of 11 variables from the survey were covered in this study. The “Documentation of client data on a mobile device saves working time” variable was used as an outcome measure. To understand what kind of factors are connected to work time savings, the following variables were used: “Age,” “Work experience,” “Workplace,” “Experience in using EHR/CIS,” “Grade for EHR/CIS,” “Most common place to document client data on a mobile device,” “Received sufficient training to document client data on a mobile device,” “Documentation of client data on a mobile device is easy,” “Documentation of client data on a mobile device reduces the need to memorize,” and “I can document everything I need on a mobile device.” A total of 9 variables were recoded in the analysis and 2 variables were included as a continuous variable. The 5-point Likert scale was specified in 5 different variables as follows: 1=fully agree, 2=agree, 3=neither agree nor disagree, 4=disagree, and 5=fully disagree. To streamline the focus on the phenomena of interest and to ensure an adequate number of respondents in all categories, the response options were recoded as follows: 1 or 2=yes and 3-5=no. The included variables are presented in Table 1.

**Table 1:** The included variables in the analysis.

Variable	Item in the survey	Response options	Coded in the analysis
Age	Year of birth	1957-2003	18-34 years, 35-54 years, and 55-65 years
Work experience	How long have you worked as a practical nurse (or equivalent)?	1=under 1 year, 2=1-2 years, 3=3-5 years, 4=6-10 years, 5=11-15 years, 6=16-20 years, and 7=over 20 years	1-3=0-5 years, 4=6-10 years, 5=11-15 years, 6=16-20 years, and 7=21 years or more
Workplace	The place of the main employment	The 3 largest places of the main employment were included in the study: 1=hospital-based home care, 2=residential care home, and 3=home care	1 or 2=service housing and 3=home care
Experience in using EHR^a^/CIS^b^	How experienced do you consider yourself to be as an EHR/CIS user?	The answer options were rated from 1=beginner to 5=highly experienced	Included as a continuous variable
Grade for EHR/CIS	How would you rate the EHR/CIS you use on a mobile device?	On a scale from 4 to 10, with 4 being the lowest score and 10 being the highest score	Included as a continuous variable
Most common place to document client data on a mobile device	What is the most common place to document client data on a mobile device?	1=next to the patient, 2=in a public area (eg, corridor), 3=on the streets, 4=at the office/nurses’ office, 5=in one’s car, 6=in the public transport, and 7=other	1=next to the patient, 2=in a public area, 4=at the (nurses’) office, 5=in one’s car, and 3,6,7=other
Received sufficient training to document client data on a mobile device	I have received sufficient training to document client data on a mobile device	5-point Likert scale^c^	Binary variables: 1 or 2=yes and 3-5=no
Documentation of client data on a mobile device is easy	Documentation of client data on a mobile device is easy	5-point Likert scale	Binary variables: 1 or 2=yes and 3-5=no
Documentation of client data on a mobile device saves working time	Documentation of client data on a mobile device saves working time.	5-point Likert scale	Binary variables: 1 or 2=yes and 3-5=no
Documentation of client data on a mobile device reduces the need to memorize things	Documentation of client data on a mobile device reduces the need to memorize things.	5-point Likert scale	Binary variables: 1 or 2=yes and 3-5=no
I can document everything I need on a mobile device	I can document everything I need on a mobile device.	5-point Likert scale	Binary variables: 1 or 2=yes and 3-5=no

^a^EHR: electronic health record.

^b^CIS: client information system.

^c^The 5-point Likert scale was specified as follows: 1=fully agree, 2=agree, 3=neither agree nor disagree, 4=disagree, and 5=fully disagree.

### Data Analysis

The data were analyzed using the statistical software SPSS Statistics version 29.0.0.0 (IBM, Inc.). The characteristics of the study participants were described using n (%). A binary logistic regression analysis was conducted to examine the association between independent and dependent variables. The “Documentation of client data on a mobile device saves working time” item was used as a dependent variable and 10 items were used as independent variables in the analysis. In establishing a model for the relationship between independent and dependent variables, we first tested the significance of each independent variable individually according to the Wald *F* test. Based on the *P* values (*P*<.05) of the Wald *F* test, the items “Age” and “I can document everything I need on a mobile device” were excluded from the regression analysis model. We included 8 other independent variables one by one in the model using a forward stepwise selection method. At each step, variables were chosen for the final model according to their effect on the model’s goodness-of-fit measure, Nagelkerke *R*^2^ (*R*^2^_N_), and *P* values of the Wald *F* test. The fully adjusted model included 7 independent variables, including “Experience in using EHR/CIS,” “Work experience,” “Workplace,” “Grade for EHR/CIS,” “Most common place to document client data on a mobile device,” “Documentation of client data on a mobile device is easy,” and “Documentation of client data on a mobile device reduces the need to memorize.” The “Received sufficient training to document client data on a mobile device” item was omitted from the final model because it was no longer statistically significant (*P*=.08) after adjusting the final variable to the model. The fully adjusted model’s *R*^2^_N_ was 0.372. Variance inflation factor was used to secure a model without multicollinearity: the values indicated low correlation, which is acceptable in a regression model. The results of the fully adjusted regression analysis model are presented with *P* values, variance inflation factor, odds ratios, and their 95% CIs in Table 4.

### Ethical Considerations

We considered ethical issues related to different phases of this study. Ethical approval for the study was provided by the Finnish Institute for Health and Welfare THL/1206/6.02.01/2022. Study participants were offered written information on the research and data processing in a cover letter and privacy notice [46]. Participants did not receive any compensation for their participation in the study. The research group has been committed to protecting the anonymity of the participants throughout the study process.

## Results

### Characteristics of the Mobile Device Users

Of the total of 1014 practical nurses who used EHRs or CISs on a mobile device, nearly one-half (471/1014, 46.45%) fell within the age range of 35-54 years. Additionally, there was a relatively high proportion of participants who were at least 55 years old. The work experience among participants was diverse and evenly distributed. For example, a portion of practical nurses (195/1014, 19.23%) had 0-5 years of experience as a practical nurse or equivalent, while others had worked for 21 years or more (238/1014, 23.47%). The majority of mobile device users (706/1014, 69.63%) were employed in home care, with the remainder working in service housing. Nearly half of the mobile device users (458/1014, 45.17%) rated their experience of using EHRs or CISs at level 4 (on a scale of 1 to 5, where 1 represents a beginner and 5 represents highly experienced). Only 4 practical nurses rated themselves as beginners in using EHRs/CISs. Additionally, the majority of practical nurses assessed the EHR/CIS system used via a mobile device as good (364/1014, 35.90%) or satisfactory (271/1014, 26.73%; Table 2).

**Table 2:** Characteristics of the study participants (n=1014).

Characteristics		Value, n (%)
**Age (years)**		
	18-34	156 (15.38)
	35-54	471 (46.45)
	55-65	387 (38.17)
	Missing data	0 (0)
**Work experience (years)**		
	0-5	195 (19.23)
	6-10	232 (22.88)
	11-15	220 (21.70)
	16-20	129 (12.72)
	21 or more	238 (23.47)
	Missing data	0 (0)
**Workplace**		
	Service housing	308 (30.37)
	Home care	706 (69.63)
	Missing data	0 (0)
**Experience using EHR^a^/CIS^b^**		
	1 (beginner)	4 (0.39)
	2	47 (4.64)
	3	287 (28.30)
	4	458 (45.17)
	5 (highly experienced)	218 (21.50)
	Missing data	0 (0)
**Grade for EHR/CIS**		
	10 (Excellent)	21 (2.07)
	9 (Very good)	110 (10.85)
	8 (Good)	364 (35.90)
	7 (Satisfactory)	271 (26.73)
	6 (Moderate)	140 (13.81)
	5 (Adequate)	90 (8.88)
	4 (Fail)	14 (1.38)
	Missing data	4 (0.39)

^a^EHR: electronic health record.

^b^CIS: client information system.

### Practical Nurses’ Experiences of Documenting Client Data on a Mobile Device

The most prevalent location for documenting client data on a mobile device was next to the client (537/1014, 52.96%). Some practical nurses also documented client data in alternative settings such as in the car, at the office, or in public areas. Overall, mobile device users expressed relatively high satisfaction with the training they received for documenting client data on a mobile device (661/1014, 65.19%). The majority of mobile device users (648/1014, 63.91%) found it easy to document client data on a mobile device. Additionally, two-thirds of practical nurses (667/1014, 65.78%) reported that documenting client data on a mobile device saved them time. Furthermore, a vast majority of mobile device users (785/1014, 77.42%) agreed that documenting client data on a mobile device reduced the need to rely on memory. Less than one-half of the participants (418/1014, 41.22%) agreed that they could document everything they need on a mobile device (Table 3).

**Table 3:** Practical nurses’ experiences of documenting client data on a mobile device (n=1014).

Variable		Value, n (%)
**Most common place to document client data on a mobile device**		
	Next to the client	537 (52.96)
	In a public area (eg, corridor)	135 (13.31)
	At the (nurses’) office	133 (13.12)
	In one’s car	175 (17.26)
	Other	31 (3.06)
	Missing data	3 (0.30)
**Received sufficient training to document client data on a mobile device**		
	No	350 (34.52)
	Yes	661 (65.19)
	Missing data	3 (0.30)
**Documentation of client data on a mobile device is easy**		
	No	361 (35.60)
	Yes	648 (63.91)
	Missing data	5 (0.49)
**Documentation of client data on a mobile device saves working time**		
	No	343 (33.83)
	Yes	667 (65.78)
	Missing data	4 (0.39)
**Documentation of client data on a mobile device reduces the need to memorize things**		
	No	222 (21.89)
	Yes	785 (77.42)
	Missing data	7 (0.69)
**I can document everything I need on a mobile device**		
	No	594 (58.58)
	Yes	418 (41.22)
	Missing data	2 (0.20)

### Factors Associated With Work Time Savings When Using EHRs/CISs on a Mobile Device

Several factors were associated with work time savings when using EHRs/CISs on a mobile device (Table 4). Experience of using EHRs/CISs (*P*<.001), work experience (*P*<.001), the workplace (*P*=.004), the grade given for the EHRs/CISs (*P*<.001), the statements “Documentation of patient data on a mobile device is easy” (*P*<.001) and “Documentation of patient data on a mobile device reduces the need to memorize things” (*P*<.001) had statistically significant associations with work time savings.

**Table 4:** The results of the fully adjusted logistic regression analysis model for the practical nurses’ experience of work time savings when using EHRs^a^/CISs^b^ on a mobile device.

Variable		Odds ratio (95% CI)	*P* value	Variance inflation factor
Experience in using EHR/CIS		1.59 (1.30-1.94)	*<.001* ^c^	1.09
**Work experience (years)**			*<.001*	1.03
	0-5	2.41 (1.43-4.07)	*.001*	
	6-10	1.34 (0.84-2.13)	.23	
	11-15	1.03 (0.64-1.67)	.89	
	16-20	0.52 (0.31-0.87)	*.01*	
	21 or more	1^d^	—^e^	
**Workplace**			*.004*	1.18
	Home care	1.95 (1.23-3.07)		
	Service housing	1		
Grade for EHR/CIS, on a scale of 4-10		0.76 (0.66-0.89)	*<.001*	1.40
**The most common place to document client data on a mobile device**			.08	1.17
	Next to the client	1.66 (0.96-2.88)	.07	
	In a public area (eg, corridor)	2.33 (1.27-4.25)	*.006*	
	In one’s car	1.84 (0.96-3.53)	.07	
	Other	2.09 (0.73-5.94)	.17	
	At the (nurses’) office	1	—	
**Documentation of client data on a mobile device is easy**			*<.001*	1.44
	Yes	3.05 (2.14-4.34)		
	No	1		
**Documentation of client data on a mobile device reduces the need to memorize things**			*<.001*	1.20
	Yes	4.10 (2.80-6.00)		
	No	1		

^a^EHR: electronic health record.

^b^CIS: client information system.

^c^The level of statistical significance was set at *P*<.05 (italicized).

^d^Comparison group.

^e^Not applicable.

Practical nurses who considered themselves to be more experienced EHR/CIS users were more likely to perceive work time savings. Participants who had worked 0-5 years as a practical nurse were 2.41 times more likely to experience work time savings compared with those who had worked 21 years or more. Practical nurses who had worked 16-20 years had a lower likelihood of experiencing work time savings than those who had worked for 21 years or more. Furthermore, practical nurses who worked in home care settings were 1.95 times more likely to report work time savings compared with those participants who worked in service housing. Giving a lower grade for EHRs/CISs was associated with a lower likelihood of experiencing work time savings. Participants who documented client data in a public area were 2.33 times more likely to experience work time savings compared with those who documented client data at the (nurses’) office. Moreover, those practical nurses who reported that the documentation of client data on a mobile device was easy were 3.05 times more likely to experience work time savings compared with others. Practical nurses who reported that the documentation of client data on a mobile device reduced their need to memorize things were 4.10 times more likely to experience work time savings compared with those who did not find mobile devices helpful in memorizing things.

## Discussion

### Principal Findings

The aim of the study was to analyze practical nurses’ experiences of using EHRs/CISs on a mobile device in their daily practice. Our study findings indicate that practical nurses generally had positive experiences when documenting client data on a mobile device. Two-thirds of the participants perceived mobile devices as effective tools in their daily practice, as they facilitated time savings in their work. The study revealed that a vast majority of the participants agreed that using EHRs/CISs on a mobile device reduced the need to memorize things. However, participants were less inclined to agree with the statement that they could document everything they needed on a mobile device.

Additionally, our study examined factors associated with work time savings when practical nurses used EHRs/CISs on a mobile device. Factors such as experience with the EHRs/CISs, work experience, workplace, the grade awarded for the EHRs/CISs, and responses to statements such as “Documentation of patient data on a mobile device is easy” and “Documentation of patient data on a mobile device reduces the need to memorize things” were all found to be associated with practical nurses’ experiences of work time savings.

### Limitations

This study has several limitations. First, the response rate of 7.16% (3866/54,030) was relatively low, which is common for web-based and lengthy surveys [47], especially those aimed at health care professionals [48]. Additionally, incorrect email addresses due to job changes or other reasons, as well as nonopened survey emails, may have further contributed to the low response rate. Therefore, the actual response rate might have been higher if calculated only for those who received and opened the survey invitation. Eventually, 3866/4533 (85.29%) survey clicks resulted in respondents completing the survey. However, it is worth noting that data collection occurred during a national industrial action organized by the trade unions, which could have complicated survey participation. Additionally, various work-related factors that practical nurses encounter in their daily routines, such as time constraints and interruptions, may have influenced survey response rates, especially considering that many union members use their work email as their contact information. Furthermore, the utilization of the convenience sampling method may restrict the generalizability of the results. However, the age distribution of the respondents mirrored that of individuals affiliated with national trade unions [49]. Additionally, the survey was available in both of Finland’s official languages, Finnish and Swedish, potentially encouraging speakers of both languages to participate.

Second, while practical nurses are a common occupational group in Finland, their title may be less recognized in other regions worldwide. Indeed, long-term care may be provided by health care professionals with various occupational titles internationally. Nonetheless, we propose that the findings of our study can be applied to other nursing professions, such as registered nurses and health care assistants, who use mobile devices as documentation tools in their daily practice. Furthermore, it is important to acknowledge that Finland has a long-standing history of extensively using ICT tools in health care [5]. Moreover, Finland ranks among the global leaders in mobile data usage [50]. Consequently, the findings of this study may be particularly relevant and applicable to countries with similar levels of ICT development.

Third, the survey was customized for practical nurses in Finland for the first time, including the questions related to mobile device use. Given the low proportion of missing data, we can assume that respondents understood the various items of the instrument relatively well. Before distribution to participants, the instrument underwent pretesting with 20 practical nurses.

In future studies, it would be beneficial to investigate work time savings among users of specific EHR/CIS brands, as the grading of the system by respondents was strongly correlated with experiencing work time savings. Additionally, research should explore specific work environments, such as home care and service housing. Hence, conducting a subgroup analysis separately for practical nurses working in home care and service housing would be a valuable addition to future studies. Another important research avenue would be to explore the barriers that practical nurses may encounter when documenting next to the patient using a mobile device.

### Comparison With Prior Work

To the best of our knowledge, this study marks the inaugural exploration of practical nurses’ experiences regarding their use of EHRs/CISs on a mobile device. Our primary focus was to investigate whether the use of EHRs/CISs on a mobile device contributes to time savings for practical nurses, as well as to identify the factors associated with such savings. In the health care sector, saving work time is crucial because nursing professionals are tasked with a multitude of responsibilities in their daily practice. It is essential for them to have more time available for direct patient care and to minimize the time spent on indirect patient care activities, such as documentation [21].

This study revealed that two-thirds of practical nurses working in home care or service housing experienced work time savings when using EHRs/CISs on a mobile device. Comparable findings of work time savings have also been documented in previous studies involving health care professionals [14,20,21]. This study revealed that documenting client data in a public area, such as a corridor in a housing service, was over 2 times more likely to result in work time savings compared with documenting at the nurses’ office, where computers are typically available. However, it is important to note that documenting sensitive client data on mobile devices in a public area may pose increased security risks, such as the potential loss or theft of the mobile device [23]. Therefore, mobile technology tools should incorporate essential security features, and organizations should establish clear policies regarding the management of mobile devices [51].

According to our study findings, work experience was linked to work time savings when using EHRs/CISs on a mobile device. Participants who had worked 0-5 years as practical nurses or in equivalent roles were over 2 times more likely to experience work time savings compared with those who had worked for over 21 years. We speculate that practical nurses with less work experience may perceive work time savings more frequently because they are accustomed to working with new technologies in their daily practice, and they may have received more recent orientation on using mobile devices. It is interesting to note that, in our analysis, age was not found to be significantly associated with work time savings when using EHRs/CISs on a mobile device. However, age may influence perceptions regarding the use of mobile devices. Findings from a previous study [52] have suggested that older nurses are less inclined to use smartphones or acknowledge their benefits in acute care settings.

Additionally, our study revealed that practical nurses working in home care settings were nearly two times as likely to report work time savings compared with those working in service housing. This finding is unsurprising, considering that home care relies on mobility and necessitates the use of ICT tools directly at patients’ homes [22]. This environment naturally fosters the integration of mobile technology into the daily practices of health care workers. An essential prerequisite for realizing the benefits of mobile technology is seamless integration with the existing information systems [18], such as EHRs/CISs. It could be hypothesized that mobile devices contribute to work time savings for practical nurses, especially in home care settings, by facilitating the documentation of client data immediately after completing daily tasks [13], such as next to the client. However, although practical nurses in this study often documented data next to the client, it was not identified as a statistically significant factor for work time savings. The immediacy afforded by mobile devices, allowing users to document client data promptly after interacting with the client, can alleviate the burden of memorization for health care professionals. According to our study findings, practical nurses who perceived that the documentation of client data reduced the need to rely on memory were 4 times more likely to report work time savings compared with those who did not find mobile devices helpful in reducing the need to memorize things.

Our study findings revealed that practical nurses who found the documentation of client data on a mobile device to be easy were over 3 times more likely to experience work time savings compared with those who did not find mobile devices easy to use. Furthermore, Zhang et al [53] discovered that nursing professionals in home care settings perceived mobile devices to be useful if the tools are easy to use. Overall, while usability issues related to health information systems, including EHRs, are widely recognized [2,54], much of the existing data are centered around the use of these systems on computers. However, it is important to note that using EHRs/CISs specifically on mobile devices may present additional challenges for social and health care professionals. For example, previous studies have indicated that mobile devices may be difficult to use, too small for daily practice [16], may not function properly at all times [13], and could be unstable due to potential internet connection problems [18,22].

In this study, the grade provided by respondents for the EHRs/CISs on a mobile device emerged as a factor associated with work time savings. Specifically, a lower grade for the EHRs/CISs was linked to a reduced likelihood of experiencing work time savings. As the grade for the EHRs/CISs may reflect user satisfaction to some extent, this finding underscores the significance of prioritizing user satisfaction regarding practical nurses’ use of EHRs/CISs on mobile devices. User satisfaction has indeed garnered significant attention in previous studies [9,15], and its impact extends beyond work time savings. According to Hsiao and Chen [9], user satisfaction influences nurses’ intention to continue using information systems on mobile devices, and perceived usefulness is often intertwined with user satisfaction. Furthermore, the quality of the information system and support from managers have been identified as significant predictors of user satisfaction [15], as well as technology adoption in general [18]. It is important to highlight that health care professionals who are more experienced users of information systems may offer valuable suggestions for improvements [9], underscoring the importance of involving these users in the development of EHRs/CISs to ensure user satisfaction with the system interfaces.

When assessing potential work time savings, it is crucial to take into account practical nurses’ experiences with using EHRs/CISs. Our study results indicate that practical nurses with more experience in using EHRs/CISs were more likely to experience work time savings. Similarly, Villalba-Mora et al [26] discovered that health care professionals who frequently used health information technologies such as EHRs perceived these tools to be more useful. Additionally, previous experience with digital technologies is significant, as it aids health care professionals in integrating mobile devices into their daily practices [18].

### Conclusions

This study contributes to the existing literature on the use of EHRs/CISs on a mobile device by practical nurses in their daily practice, as well as factors associated with work time savings. Our findings indicate that two-thirds of practical nurses perceived mobile devices as beneficial in home care and service housing settings, as they reported that documenting client data on a mobile device saved their working time. Experience in using EHRs/CISs, work experience, workplace, grade given for the EHRs/CISs, and perceptions regarding the ease of documentation and reduction in the need to memorize were all significantly associated with practical nurses’ experiences of work time savings. Based on our findings, we recommend that special attention should be directed toward mobile device users who are less experienced in using EHRs/CISs or do not find mobile devices easy to use. Organizations should provide comprehensive orientation and regular education to health care professionals on the use of EHRs/CISs on mobile devices. Additionally, user satisfaction is a crucial aspect to consider in achieving work time savings among health care professionals who use EHRs/CISs on a mobile device, as demonstrated by our findings. Practical nurses who rated their EHRs/CISs more favorably were more likely to experience work time savings. Therefore, we suggest that end users, particularly those with more experience in using EHRs/CISs, should be involved in the development of EHRs/CISs to ensure better user satisfaction of system interfaces.

## Abbreviations

CIS: client information system
EHR: electronic health record
ICT: information and communication technology
STePS: Monitoring and Assessment of Social Welfare and Health Care Information System Services

## Data Availability

The data collected and analyzed during this study are not publicly available due to coregistratorship between the University of Eastern Finland and the Finnish Institute for Health and Welfare, the controllers of the data.
